# Characterization of Begomoviruses Sampled during Severe Epidemics in Tomato Cultivars Carrying the *Ty-1* Gene

**DOI:** 10.3390/ijms19092614

**Published:** 2018-09-03

**Authors:** Covadonga Torre, Livia Donaire, Cristina Gómez-Aix, Miguel Juárez, Michel Peterschmitt, Cica Urbino, Yolanda Hernando, Jesús Agüero, Miguel A. Aranda

**Affiliations:** 1 Abiopep S.L., Departamento de I + D + i, Parque Científico de Murcia, Ctra. de Madrid, Km 388, Complejo de Espinardo, Edf. R, 2°, 30100 Murcia, Spain; ctorre@abiopep.com (C.T.); cgaix@abiopep.com (C.G.-A.); yh.saiz@abiopep.com (Y.H.); jaguero@abiopep.com (J.A.); 2 Centro de Edafología y Biología Aplicada del Segura (CEBAS)-CSIC, Departamento de Biología del Estrés y Patología Vegetal, P.O. Box 164, 30100 Murcia, Spain; ldonaire@cebas.csic.es; 3 Escuela Politécnica Superior de Orihuela, Universidad Miguel Hernández de Elche, Ctra. de Beniel, Km 3.2, 03312 Alicante, Spain; miguel.juarez@umh.es; 4 Centre de Coopération Internationale en Recherche Agronomique Pour le Développement (CIRAD), UMR-BGPI, Equipe Interactions Virus-Insecte-Plante, TA A-54/K, Campus International de Baillarguet, CEDEX 5, 34398 Monptellier, France; michel.peterschmitt@cirad.fr (M.P.); cica.urbino@cirad.fr (C.U.)

**Keywords:** geminivirus, resistance, resistance breaking, TYLCV, virus evolution

## Abstract

*Tomato yellow leaf curl virus* (TYLCV, genus Begomovirus, family Geminiviridae) is a major species that causes a tomato disease for which resistant tomato hybrids (mainly carriers of the *Ty-1*/*Ty-3* gene) are being used widely. We have characterized begomoviruses severely affecting resistant tomato crops in Southeast Spain. Circular DNA was prepared from samples by rolling circle amplification, and sequenced by massive sequencing (2015) or cloning and Sanger sequencing (2016). Thus, 23 complete sequences were determined, all belonging to the TYLCV Israel strain (TYLCV-IL). Massive sequencing also revealed the absence of other geminiviral and beta-satellite sequences. A phylogenetic analysis showed that the Spanish isolates belonged to two groups, one related to early TYLCV-IL isolates in the area (Group 1), and another (Group 2) closely related to El Jadida (Morocco) isolates, suggesting a recent introduction. The most parsimonious evolutionary scenario suggested that the TYLCV isolates of Group 2 are back recombinant isolates derived from TYLCV-IS76, a recombinant virus currently predominating in Moroccan epidemics. Thus, an infectious Group 2 clone (TYLCV-Mu15) was constructed and used in in planta competition assays against TYLCV-IS76. TYLCV-Mu15 predominated in single infections, whereas TYLCV-IS76 did so in mixed infections, providing credibility to a scenario of co-occurrence of both types of isolates.

## 1. Introduction

The Tomato yellow leaf curl disease (TYLCD) is one of the most devastating for tomato crops in the Mediterranean basin, as well as in many tropical and subtropical areas, producing losses in crops of up to 100% in certain areas [1,2]. TYLCD is caused by viruses of the genus Begomovirus (family Geminiviridae), including up to 10 different species [3,4,5]. *Tomato yellow leaf curl virus* (TYLCV) is one of the most widely cited species and includes seven different strains, of which Mild (TYLCV-Mld) and Israel (TYLCV-IL) are prevalent in Mediterranean countries [3,6]. The TYLCV genome is composed of a circular single-stranded DNA molecule of about 2.8 kb that includes six ORFs, two in the positive (viral) sense and four in the complementary sense of the genome [7,8]. The viral DNA is encapsidated in twined icosahedral virions. TYLCV is transmitted by the whitefly *Bemisia tabaci* in a persistent and circulative manner [9], and seed transmission has been reported [10].

TYLCD-affected plants were identified for the first time in Spain in 1992, and Tomato yellow leaf curl Sardinia virus (TYLCSV) was described as the causal agent during that time [11,12]. The first cases of TYLCV documented in Spain occurred five years later in 1997, corresponding to TYLCV-Mld isolates [13,14]. The first TYLCV-IL cases were described in 2003 [15], although a later study showed that isolates of this strain were present in the fields since 1999 [16]. The coexistence of these viruses could probably lead to the appearance of the two recombinant species described in Spain: *Tomato yellow leaf curl Málaga virus* (TYLCMaV), a recombinant between TYLCSV and TYLCV-Mld, described in 2002, but present since 1999 [17] and *Tomato yellow leaf curl Axarquía virus* (TYLCAxV), a recombinant between TYLCSV and TYLCV-IL that first appeared in 2000 [18]. However, the exclusive identification of TYLCV-IL isolates from 2003 suggested a complete displacement of the rest of the agents involved in TYLCD in tomato, establishing this as the dominant and representative virus in Spanish epidemics in this crop [16,19].

Traditionally, the control of TYLCD has been based on the control of the viral vector, which can be expensive and often inefficient, so that the use of resistant cultivars has become the best alternative for disease control [2,20]. Tomato is highly susceptible to the viruses that induce TYLCD, so all efforts to find resistance sources have been focused on wild tomato relatives [2,20]. Up to six resistance genes have been mapped (*Ty-1* to *Ty-6*), all of them from wild species [21]. Among them, *Ty-1* is the most used in commercial cultivars. This gene was identified in *Solanum chilense* accession LA1969 [22] and its recent mapping showed that it was allelic to *Ty-3* (*S. chilense*, accession LA1932) [23,24]. *Ty-1*/*Ty-3* encodes an RNA-dependent RNA polymerase and the mechanism of resistance seemed to be based on transcriptional gene silencing associated with an increase in viral genome methylation [21,24]. In plants carrying these resistance alleles, viral replication still occurs, although at a lower level than in susceptible plants [25,26,27], so that cultivars in the market are partially resistant to TYLCD [28].

The main mechanisms of genetic variation of viruses are mutation and recombination [29]. For geminiviruses, recombination seems to have had a fundamental role in the generation of new variants with unique biological features [30]. In this regard, a new recombinant between TYLCV and TYLCSV, called TYLCV-IS76, has been recently described in Morocco associated with *Ty-1* resistant tomato plants bearing TYLCD symptoms. TYLCV-IS76-like isolates increased in frequency until the complete displacement of their parental viruses in 2012 in Morocco, coincident with the widespread use of tomato *Ty-1/Ty-3* cultivars [31]. Analyses of virus accumulation under experimental conditions in resistant and susceptible tomato plants showed that TYLCV-IS76 had greater in planta fitness than its parental viruses, providing an explanation for the field observations [27]. A hypothetical introduction of TYLCV-IS76-like isolates in Spain thus represents a risk worth evaluating.

During recent years, we have been observing aggressive TYLCD symptoms in plants carrying the *Ty-1*/*Ty-3* resistance gene in tomato crops in Murcia (Southeast Spain), similar to those described in Morocco [31]. Murcia is one of the main Spanish tomato-producing areas. We collected samples for two consecutive years, 2015 and 2016, in Águilas and Mazarrón, the two main areas for tomato cultivation in Murcia. The sequencing of rolling circle amplification (RCA) products from these samples, followed by population genetics and phylogenetic analyses, showed that two types of isolates cocirculated in the epidemics, both belonging to the Israel strain, one including isolates similar to the ones described previously in the area, and another group that was phylogenetically related to recently-described Moroccan isolates. However, unlike TYLCV-IS76, these did not show any evidence of recombination. An infectious clone (TYLCV-Mu15) was prepared for one isolate of the second group, and its in planta fitness was compared with that of TYLCV-IS76 [27]; in single infections in resistant tomato plants, TYLCV-Mu15 accumulation was significantly higher than that of TYLCV-IS76, while in mixed infections, TYLCV-IS76 predominated. Therefore, our results will help to understand changes in the populations of viruses associated with TYLCD in Spain. 

## 2. Results

### 2.1. Aggressive Tomato Yellow Leaf Curl Virus (TYLCV) Epidemics in Resistant Tomato Crops in Murcia (Spain)

**Figure 1:**
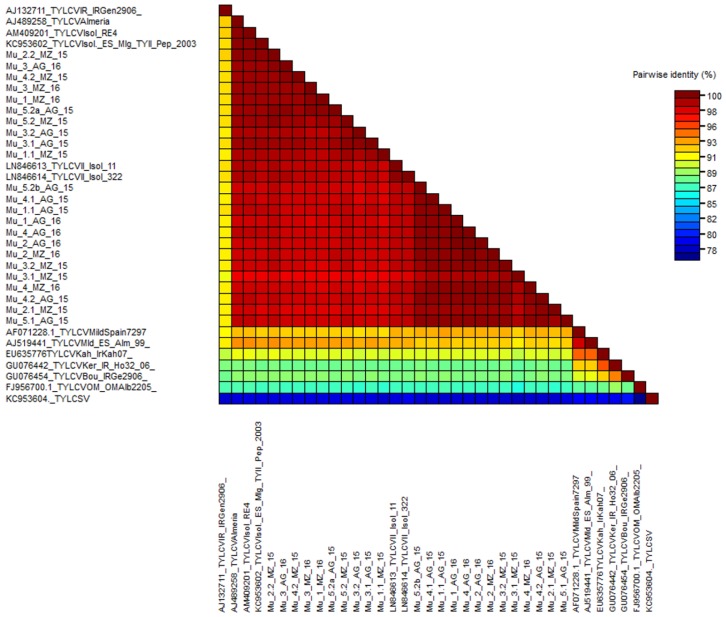
Genome-wide pairwise identity color matrix constructed with sequences determined in this article and representatives of the different strains of TYLCV calculated by SDT v1.2 [32]. TYLCSV (Tomato yellow leaf curl Sardinia virus) was used as outgroup. TYLCV-IL: *Tomato yellow leaf curl virus* strain Israel; TYLCV-Om: *Tomato yellow leaf curl virus* strain Oman; TYLCV-Mld: *Tomato yellow leaf curl virus* strain Mild; TYLCV-Kah: *Tomato yellow leaf curl virus* strain Kahnooj; TYLCV-Bou: *Tomato yellow leaf curl virus* strain Boushehr; TYLCV-Ker: *Tomato yellow leaf curl virus* strain Kerman; and TYLCV-Ir: *Tomato yellow leaf curl virus* strain Iran.

An unusually aggressive TYLCD outbreak was noticed in tomato crops in Murcia (Southeast Spain) by the end of the 2015 summer. Tomato cultivars were TYLCV resistant (*Ty-1*/*Ty-3* gene) and yet the plant symptoms were remarkable, including severe stunting, curling of the leaves, and yellowing. Affected areas were surveyed, and 20 samples were collected, two from each surveyed plot in each location (Table 1). Total DNA was extracted from these samples; all preparations were geminivirus-positive in a preliminary PCR assay (data not shown). RCA was used to enrich preparations in circular DNA and the products were submitted to high throughput sequencing (HTS). The 20 libraries sequenced produced between 3092 and 31436 readings with an average size per reading of 300 bp. For the mapping of the sequences, the genomes of TYLCV isolates from Reunion, Morocco, Spain, and Japan were used as references (accession numbers AM409201, LN846615, LN846614, AJ0489258, and AB192965). We obtained between 26 and 8560 mapped readings per library, covering between 79 and 100% of the reference genomes (Table A1) with coverage depth average values between 2 and 693. In total, 15 consensus sequences could be reliably determined, seven from Mazarrón and eight from Águilas, having between 2780 and 2782 nucleotides corresponding to complete TYLCV genomes. BLASTn analysis of these sequences revealed a nucleotide similarity equal to or greater than 98% with other TYLCV sequences belonging to the Israel strain (TYLCV-IL). HTS sequences were further mapped against geminivirus satellite sequences, and no additional hits were identified. In 2016, a similar epidemiological situation was noticed, leading to a second sampling in the same areas. In this case the number of plots was four per geographical area and five samples were taken per plot (Table 1). Total DNA was extracted from these samples; all preparations were geminivirus-positive in a preliminary PCR assay (data not shown). Restriction digestion of RCA products and their subsequent cloning and Sanger sequencing was the strategy followed for the determination of nucleotide sequences in this case. A total of eight full-length clones were sequenced, four from Águilas and four from Mazarrón (Table 1). An identity analysis by pairs using the SDT v1.2 program [32] was carried out. In addition to the sequences determined here, the sequences of other TYLCV strains were included: Israel (AJ489258, EF060196, KC953602, LN846613, LN846614, and AB116629), Mild (AF071228 and AJ519441), Iran (AJ132711), Kahnooj (EU635776), Kerman (GU076442), Boushehr (GU076454), Oman (FJ956700), as well as a sequence of a representative of the Sardinia species (KC953604). Results showed that the isolates sequenced in this work shared nucleotide identity percentages ranging from 97.2% to 99.8% (Figure 1). The highest percentages of identity (around 97%) were from the TYLCV-IL sequences. Thus, according to the demarcation criteria for geminiviruses, which established the demarcation of species as 91% and that of strains as 94% [3], all the sequences determined here belonged to TYLCV-IL.

A striking result of the SDT analysis was the grouping of the isolates into two groups, apparently independent of the year and area sampled. There was a group of 10 isolates (Group 1) that included Mu 2.2 Mz: 15, Mu 1.1 Mz: 15, Mu 5.2 Mz: 15, Mu 3.2 Ag: 15, Mu 3.1 Ag: 15, Mu 4.2 Mz: 15, Mu 3 Ag: 16, Mu 5.2a Ag: 15, Mu 1 Mz: 16 and Mu 3 Mz: 16, and a group of 13 isolates (Group 2) that included Mu 3.1 Mz: 15, Mu 3.2 Mz: 15, Mu 2 Mz: 16, Mu 4 Ag: 16, Mu 2 Ag: 16, Mu 1 Ag: 16, Mu 2.1 Mz: 15, Mu 4.2Ag: 15, Mu 4 Mz: 16, Mu 1.1 Ag: 15, Mu 5.1 Ag: 15, Mu 4.1 Ag: 15, and Mu 5.2b Ag: 15. Identity by pairs was greater within each group than between the groups: between 98.6% and 99.7% for Group 1 and between 98.6% and 99.9% for Group 2, while between both groups the percentages observed were between 97.2% and 98.6%. These data agreed with an analysis based on genetic distances, which allowed for the estimation of the degree of genetic variation within and between populations [33]. The mean nucleotide distance between isolates from Group 1 was 0.008 ± 0.0005 and 0.005 ± 0.001 for Group 2, while the distance between isolates from different groups was 0.012 ± 0.001.

Based on these results it was concluded that the aggressive epidemics observed in Murcia in 2015 and 2016 in resistant tomato cultivars were associated with TYLCV isolates belonging to the Israel strain and that these could be differentiated into two groups regardless of the year and geographical area sampled.

### 2.2. Phylogenetic Analysis of the Murcia (Spain) Isolates Sampled during 2015 to 2016

**Figure 2:**
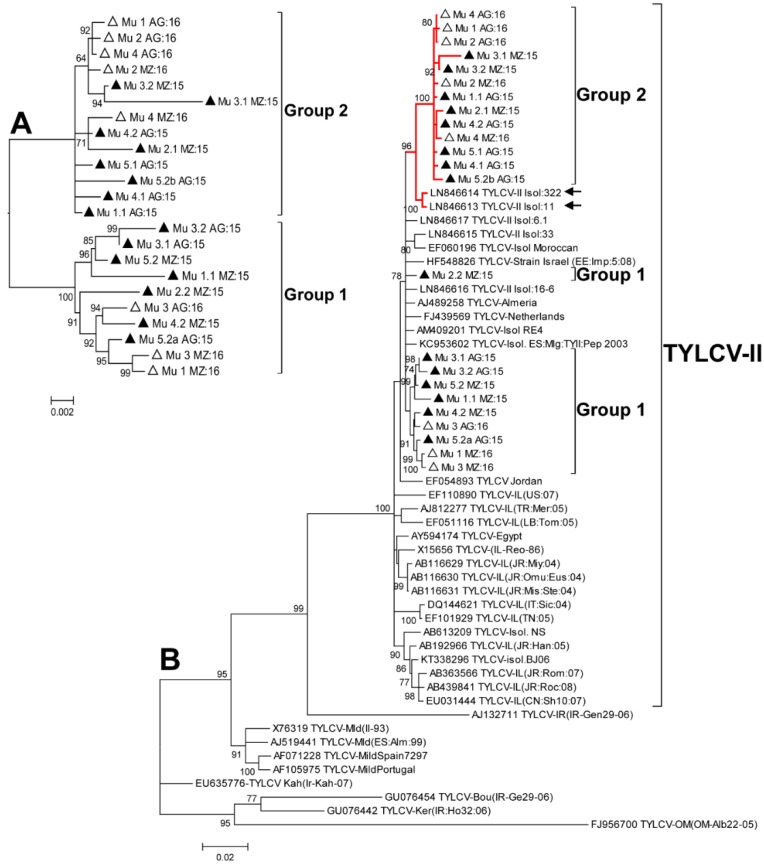
Phylogenetic analysis of full genome sequences of *Tomato yellow leaf curl virus* (TYLCV). Trees were constructed using the maximum likelihood method with 1000 bootstrap replicates applying the Tamura-Nei + G model. Branch nodes with <50% bootstrap values were collapsed and bootstrap values >70% are shown. Symbols before sequence names: Filled triangles, sequences determined in this work in 2015; empty triangles, sequences determined in this work in 2016. Sequences determined in this work are highlighted as Group 1 or Group 2. (**A**), tree constructed with the sequences determined in this work. (**B**), tree constructed with sequences of the Israel strain, the sequences determined in this work, and representatives of the other strains of TYLCV. Node corresponding to the Israel strain is highlighted. Red branches: node corresponding to group 2 and related Moroccan sequences. Arrows correspond to Moroccan sequences [31] related to Group 2. TYLCV-IL: TYLCV strain Israel; TYLCV-Om: TYLCV strain Oman; TYLCV-Mld: TYLCV strain Mild; TYLCV-Kah: TYLCV strain Kahnooj; TYLCV-Bou: TYLCV strain Boushehr; TYLCV-Ker: TYLCV strain Kerman, and TYLCV-Ir: TYLCV strain Iran.

**Figure 3:**
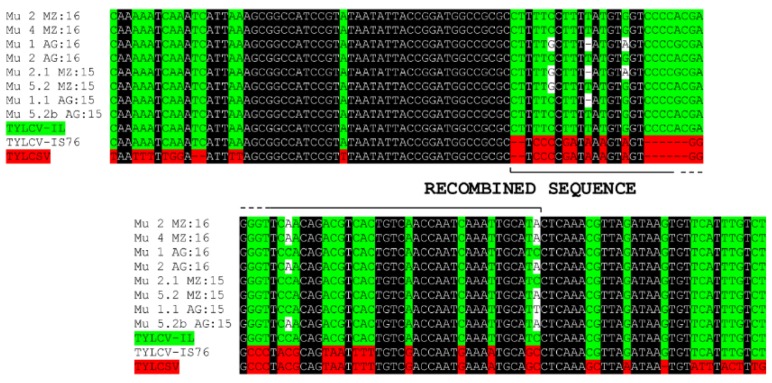
Alignment of the TYLCV and TYLCSV intergenic regions including the recombination event described for TYLCV-IS76 [31]. Sequences labeled with Mu were determined in this work and belong to the so-called Group 2. Conserved sequences are highlighted in black; those corresponding to TYLCV or TYLCSV are marked in green or red, respectively. The line delimits the recombined sequence. Sequences used for the alignment: TYLCV-IL: TYLCV strain Israel (ref. LN846614); TYLCV-IS76: TYLCV recombinant IS76 (ref. LN831187); and TYLCSV: *Tomato yellow leaf curl Sardinia virus* (ref. KC953604).

In order to understand the origin and diversification of TYLCV in Murcia during 2015 to 2016, a phylogenetic analysis was performed (Figure 2). First, we analyzed only the sequences determined in this work and this analysis showed the existence of two branches in the tree that included different isolates, independent of the year and geographic area sampled (Figure 2A), coincident with Groups 1 and 2 as determined by the population diversity analyses described above. A second phylogenetic analysis was carried out in which additional sequences from databases were included (Figure 2B). Interestingly, in this tree, Group 2 sequences appeared to be closely related to two Moroccan sequences (LN846613 and LN846614) (Figure 2B), recently described by Belabess et al. (2015) [31] for isolates sampled in the El Jadida (Morocco) region; note that these are nonrecombinant isolates [31]. To rule out a potential recombinant origin for Spanish isolates in Group 2 similar to the one originating TYLCV-IS76, both an alignment with TYLCV-IS76 and TYLCSV in the recombination region (Figure 3), as well as an analysis of the sequences using the RDP4 program (data not shown) were carried out; no recombination events were identified.

**Figure 4:**
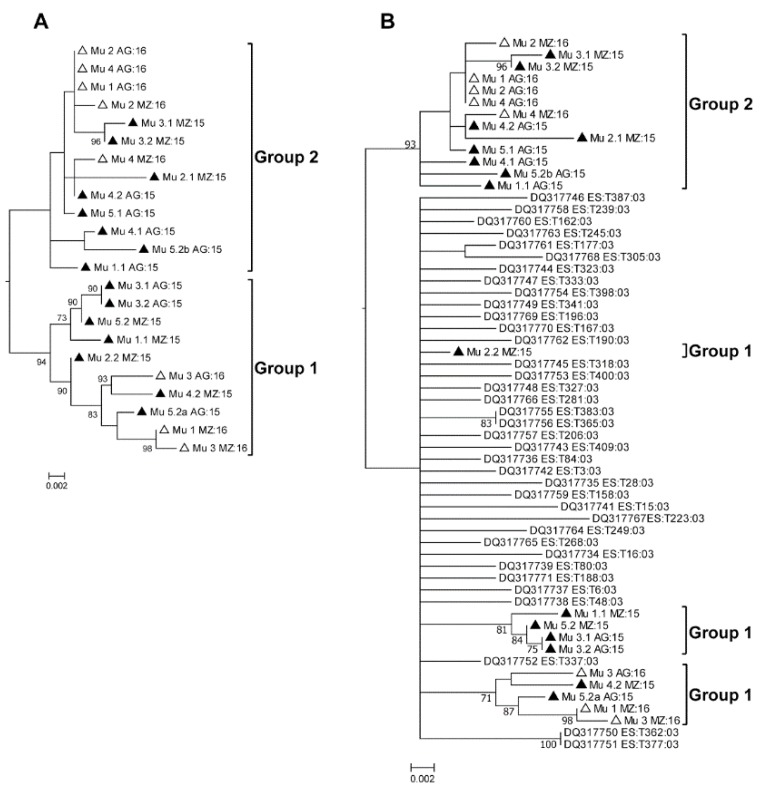
Phylogenetic analysis of partial genome sequences of *Tomato yellow leaf curl virus* strain Israel (TYLCV-IL) corresponding to the intergenic region and 5′-proximal parts of Rep and V2 ORFs, as shown in García-Andrés et al. (2007) [16]. Trees were constructed using the maximum likelihood method with 1000 bootstrap replicates and applying the Tamura-3 parameter+G model. Only bootstrap values >70% are shown. Symbols before sequence names: Filled triangles, sequences determined in 2015; Empty triangles, sequences determined in 2016. (**A**), tree constructed with the sequences determined in this work. (**B**), tree constructed with the sequences determined in this work and sequences from Spain from 2003 [16]. Murcia 2003 sequences correspond to NCBI accessions DQ317757 to DQ31777.

To analyze if the situation observed in the field could be attributed to the introduction of a new variant of the virus that was different from what had predominated in the field in previous years, a new phylogenetic analysis was carried out including sequences determined for early Murcian isolates [16] and deposited in databases. The sequences were partial and were comprised of a fragment of approximately 780 nt including part of the 5′ end of the Rep ORF (including a fragment of the overlapping region of the C4 ORF), the entire intergenic region, and part of the 5′ end of the V2 ORF (including an overlapping fragment of the 5′ end of the CP). First, only the 2015 and 2016 Murcian sequences were included in the analysis, to test whether the use of partial sequences (Figure 4A) could reproduce the results obtained with the complete sequences (Figure 2A). The topologies of the trees of Figure 2A and Figure 4A were similar, with the same isolates appearing in the same branches, validating this approach. When the sequences of early Spanish isolates [16] were included in the analysis, Group 1 isolates appeared related to a branch along with the early Spanish isolates, but Group 2 appeared in an independent branch (Figure 4B). These analyses suggested that isolates of Group 1 had been in the area for a long time, while those of Group 2, closely related to El Jadida isolates (Figure 2B), had evolved or appeared in the area only recently.

**Figure 5:**
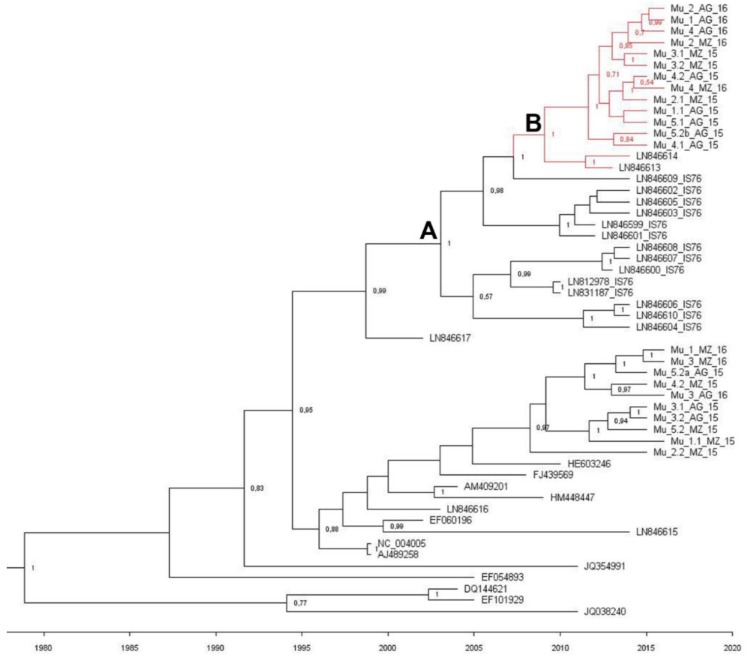
Maximum clade credibility tree reconstructed from the Spanish, Moroccan, and selected *Tomato yellow leaf curl virus* strain Israel (TYLCV-IL) nucleotide sequences dataset. The genomic region recombined in TYLSV-IS76 isolates was excluded from the alignment. Each taxon was named according to the GenBank accession number of its sequence except the taxa identified in this study, which were named with their sequence codes (Table 1). The time-scale is indicated below the tree. Node labels correspond to posterior probability support values (values below 40% are not shown). The branches supporting the El Jadida/Murcian Group 2 clade are colored in red. Node B corresponds to the most recent common ancestor of the parental genome of El Jadida/Murcian Group 2 and node A to the most recent common ancestor of TYLCV-IS76 Moroccan recombinants and El Jadida/Murcian Group 2.

We then performed a Bayesian analysis to date the diversification for TYLCV Spanish and Moroccan isolates (Figure 5). We followed the same approach as in Belabess et al. (2015) [31], including recombinant TYLCV-IS76 isolates and other sequences selected from the databases. The short TYLCSV derived region of TYLCV-IS76 recombinants was excluded from the alignment, and its cognate was similarly excluded from all the aligned sequences. The estimated mean substitution rate was 5.75 × 10^−4^ subs/site/year with a 95% highest probability density (HPD) ranging from 3.66 × 10^−4^ to 7.94 × 10^−4^, which is in agreement with other studies made for TYLCV [34]. Our results showed that Spanish Group 2 and El Jadida isolates shared a common recent ancestor around 7.1 years ago (HDP 95% 4.4–10.4 years) (node B). The age of the most recent common ancestor for the TYLCV-IS76 group and the El Jadida and Murcian Group 2 isolates (node A) was estimated to be 13.2 years (HPD 95% 9–17.8 years). The most parsimonious evolution scenario, i.e., the one that needs the minimum number of recombination events is as follows: One recombination event to generate the ancestor of all TYLCV-IS76 recombinants (ancestor A), and one reverse recombination event to generate a “nonrecombinant” TYLCV (ancestor B).

In order to understand the causes of the apparent structuring of the TYLCV population in Murcia, an analysis of the direction and intensity of the selection that acted on the different regions of the viral genome was carried out (Table 2). By estimating the number of synonymous (ds) and nonsynonymous (dn) substitutions between sequence pairs, the difference dn-ds can be determined, which if positive suggests that the gene is under positive or diversifying selection, if negative suggests that the gene is under negative or purifying selection and if it is equal to 1 the gene is under neutral selection. In general, the dn-ds differences calculated for the genes *V1*, *V2*, *C1*, *C2*, and *C4* were negative (Table 2), which suggests that these genes were under negative selection. However, for the *C3* gene, the value of the dn-ds ratio was 0.008, which suggests that this gene was under positive selection.

### 2.3. Comparing the Fitness of TYLCV-Mu 15 and TYLCV-IS76 in Susceptible and Resistant (Ty-1/Ty-3) Tomato Plants

According to the most parsimonious evolutionary scenario previously described, i.e., the one that needs the minimum number of recombination events, the TYLCV isolates of Group 2 were derived from TYLCV-IS76 based on two recombination events. With such a scenario, it is expected that TYLCV-IS76 and the back recombinant TYLCV were co-occurring in the same environment when the back recombinant TYLCV emerged. To test the credibility of this scenario we compared the biological fitness of a Group 2 isolate with that of a TYLCV-IS76 isolate [27]. Thus, the sequence Mu 5.2bAG:15 (Group 2; Figure 2A and Table A1) was used to synthesize an infectious clone. The infectious clone of TYLCV-IL previously used to determine the competitiveness of TYLCV-IS76 was also included in the comparison. From now on, each isolate will be referred to regardless of the acronym of the virus, so we will refer to IL, IS76, and Mu15 for single inoculations and to IL + IS76 and Mu15 + IS76 for mixed inoculations.

**Figure 6:**
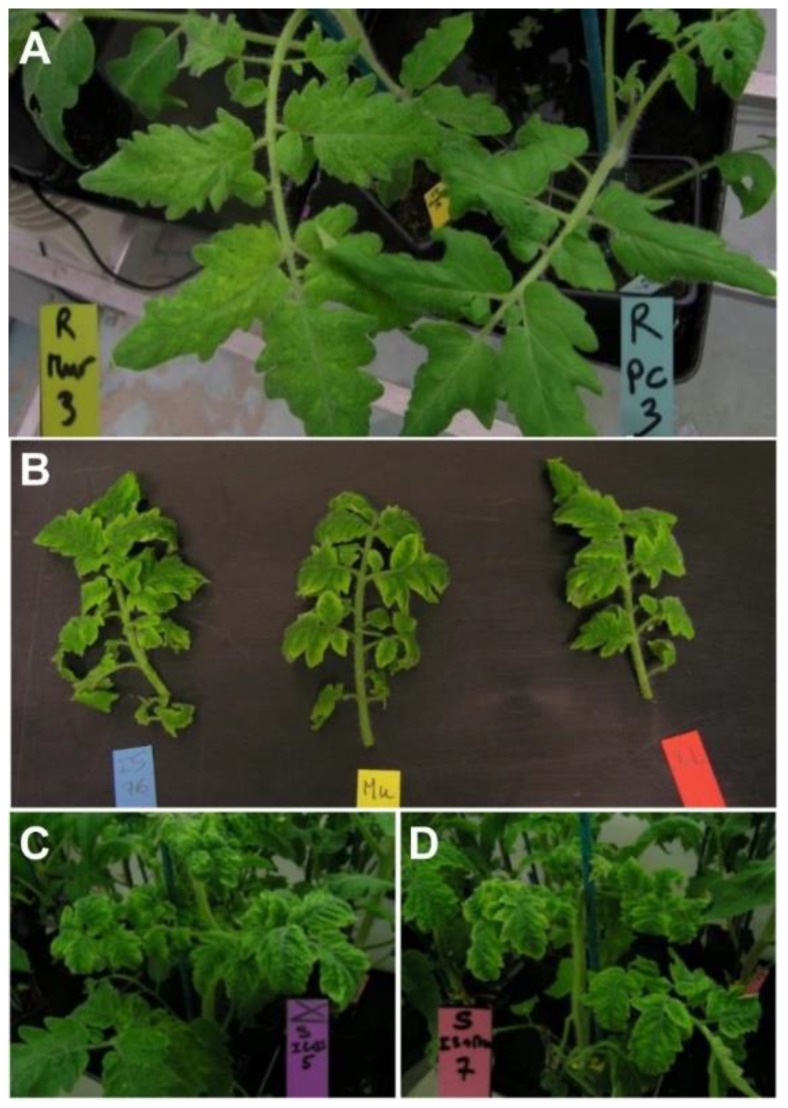
(**A**) Slight yellow mosaic in Pristyla tomato plants (resistant, *Ty-1*) agroinoculated with pTYLCV-Mu15 (**left**) compared with same plants inoculated with empty vector (**right**) at 18 days post inoculation (dpi). (**B**–**D**) Symptom expression in susceptible plants at 30 dpi; (**B**) comparison between clones in single infection, from left to right: TYLCV-IS76; TYLCV-Mu15, and TYLCV-IL; (**C**,**D**) susceptible plants mixed inoculated with TYLCV-IL + TYLCV-IS76 and pTYLCV-Mu15 + TYLCV-IS76, respectively.

**Figure 7:**
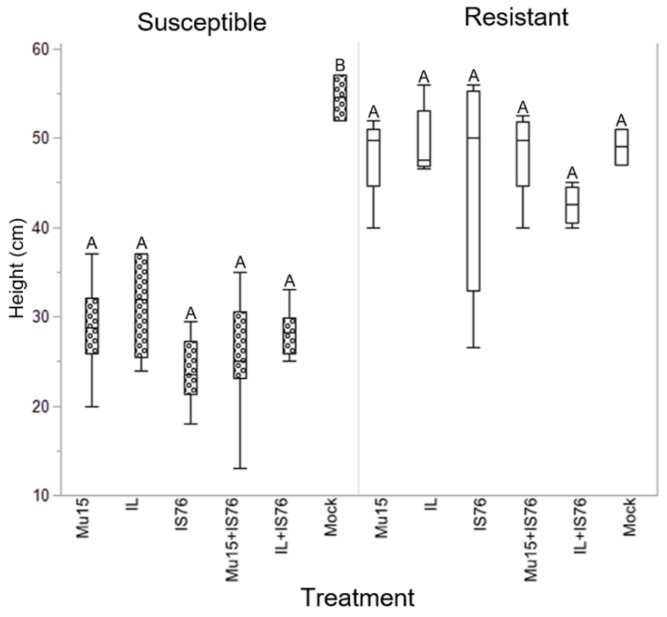
Stem measures (cm) in resistant and susceptible plants at 30 dpi, comparing the effect of the different treatments against control plants inoculated with an empty vector (Mock). Boxplot depicts the median line and delimits the 25 and 75% quantiles. Lines represent the range of values. Capital letters indicate significant differences (Kluskal–Wallis test, *p* < 0.05) where A represents no significant differences and B represents significant differences. Agroinoculation treatments: single infection, Mu15 (clone TYLCV-Mu15), IL (clone TYLCV-IL), IS76 (clone TYLCV-IS76), and mixed infections Mu15 + IS76 (a mixture of TYLCV-Mu15 and TYLCV-IS76) and IL + IS76 (a mixture of TYLCV-IL and TYLCV-IS76).

At 10 days post-inoculation (dpi) disease symptoms were already observed in 63% of the susceptible plants. At 18 dpi, 100% of susceptible plants showed typical TYLCV symptoms and some of the resistant plants inoculated with IL (one plant), Mu15 (two plants), IL + IS76 (one plant) and Mu15 + IS76 (three plants) showed a slight yellow mosaic compared to the negative controls (Figure 6A). By the end of the experiment, at 30 dpi, the expression of the symptoms in susceptible plants was similarly severe for all three clones in single (Figure 6B) or mixed infections (Figure 6C,D). In resistant plants, at 30 dpi, symptoms were no longer observed. In addition to the evaluation of symptoms, the length of the stem from the cotyledons to the apex was measured at 30 dpi; a large difference was observed between susceptible and resistant plants (Figure 7). In the susceptible plants, no significant differences were observed between the different inoculation treatments, but these differences were significant with respect to the plants inoculated with the empty vector (*p* = 0.03). In the resistant plants, either in single or in mixed infections, no significant differences were observed with respect to those inoculated with the empty vector (*p* = 0.19) (Figure 7).

**Figure 8:**
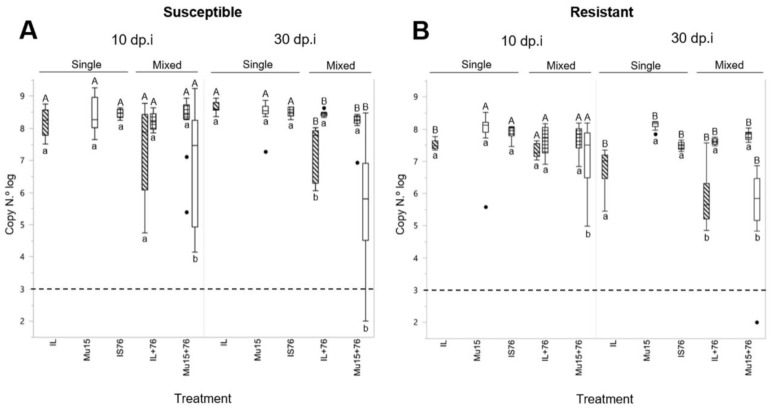
Viral DNA accumulation in susceptible (**A**) and resistant (**B**) plants at 10 and 30 dpi. DNA accumulation is expressed as the logarithm of the number of copies. Boxplot depicts the median line and delimits the 25 and 75% quantiles. Lines represent the range of values. Capital letters indicate significant differences (Kluskal–Wallis test, *p* < 0.05) in comparisons between single-infected viruses or between different viruses in mixed infections, where A represents no significant differences and B represent significant differences. Small letters indicate significant accumulation differences (Wilcoxon test, *p* < 0.05) in comparisons of the same virus between treatments, where a represent no significant differences and b represents significant differences. Boxplot patterns correspond to: White, Mu15 (TYLCV-Mu15); oblique lines, IL (TYLCV-IL); squares, IS76 (TYLCV-IS76). Agroinoculation treatments: single infection, Mu15 (clone TYLCV-Mu15), IL (clone TYLCV-IL), IS76 (clone TYLCV-IS76) and mixed infections Mu15 + IS76 (a mixture of TYLCV-Mu15 and TYLCV-IS76) and IL + IS76 (a mixture of TYLCV-IL and TYLCV-IS76).

In order to measure virus accumulation, samples were taken at 10 and 30 dpi and viral DNA was quantified by real-time PCR as in Belabess et al. (2016) [27]. In susceptible plants, in single infections, no significant differences were observed at any of the two post-inoculation times. In contrast, in mixed infections at 10 dpi, differences were already observed in the two treatments, which became significant at 30 dpi, with greater accumulation of IS76 compared to the other two clones; this difference was particularly marked in the case of coinfection with Mu15, where there were plants in which accumulation levels were below the limit of detection (Figure 8A). In resistant plants, in single infections, a tendency towards greater accumulation of Mu15 was detected, which was more evident at 30 dpi: At 10 dpi, Mu15 and IS76 accumulated at similar and significantly higher levels than IL, while at 30 dpi, Mu15 accumulated at significantly higher levels than the other two clones (Figure 8B), reaching levels of accumulation up to 10 times higher with respect to IL. Notably, this trend was reversed in mixed infections, in which coinfection of IS76 with Mu15 resulted in a decrease of Mu15 accumulation, with values significantly lower at 30 dpi (Figure 8B). This effect of mixed infections on virus accumulation was also analyzed by comparing virus clones throughout all the treatments. While IS76 accumulation levels did not show significant differences among treatments at any of the post-inoculation times, independently of the cultivar analyzed, differences were observed in the accumulation of the two other clones between treatments, especially for Mu15, where at 10 dpi significant differences were already observed in single versus mixed infections in both susceptible and resistant plants. In conclusion, TYLCV-Mu15 and TYLCV-IL accumulation was repressed in plants that were mixed infected with TYLCV-IS76; in contrast, TYLCV-Mu15 may have had a competitive advantage in singly infected resistant plants, as at 30 dpi, it accumulated at significantly higher levels than the other two isolates.

## 3. Discussion

Begomovirus identification studies are particularly efficient when combining HTS with enrichment of samples in circular DNA by RCA prior to sequencing [37,38]. Thus, we used this combination of techniques to study the unusually severe TYLCD epidemics observed in *Ty-1* tomato crops in 2015 in Murcia. Our results indicated that epidemics were associated with isolates of the TYLCV-IL strain, and ruled out the presence of other geminivirus species, such as ToLCNDV, which has been discovered recently infecting cucurbits and tomato crops in Spain [39,40], as well as the presence of betasatellites, circular DNA molecules associated with monopartite begomoviruses [41]. Both population and phylogenetic analyses supported the existence of two groups of TYLCV-IL isolates cocirculating in the epidemics regardless of the year and area sampled. Phylogenetic analysis using partial sequences suggested that isolates of Group 1 may have been present in the area for some time due to its phylogenetic relationship with early Spanish isolates. In contrast, Group 2 isolates appeared related to recently discovered Moroccan isolates from the El Jadida region. Our analyses suggested that the TYLCV isolates of Group 2 are derived from a Moroccan TYLCV-IS76 recombinant through a back recombination event, as previously suggested by Belabess et al. (2015) for the El Jadida isolates [31]. This scenario, with a single recombination event leading to TYLCV-IS76 and a back recombination to generate the El Jadida TYLCV type, is consistent with field observations and experimental results which suggest that TYLCV-IS76 emergence is a rare event [42]. The relatively higher accumulation of the Group 2 infectious clone in single infections of the *Ty-1* resistant plants may explain how the El Jadida isolates could be maintained in an environment containing TYLCV-IS76; its maintenance may have needed a low inoculation pressure to limit the percentage of co-infected plants in which its recombinant competitor was shown to have a drastic advantage. As only two isolates of this back recombinant type were characterized previously [31], it was not known if isolates of this type are competitive enough to increase in frequency in nature. Now, the discovery of sister isolates in Spain indicates that this back recombinant was indeed competitive, at least in Spain. As the accidental introduction of a new pathogen is rarely massive, it is likely that Group 2 isolates have to some extent displaced the indigenous TYLCV isolates from an initially low introduced inoculum; it is worth to recall that about 50% of the sampled tomato plants irrespective of the location and the year of sampling were of the Group 2 type. The traffic of infected material, as well as vector movements seem to be the cause of the wide distribution of TYLCD throughout the world [43,44]. In addition, infected fruits are able to serve as the inoculum source for *Bemisia tabaci* at least under experimental conditions [45]. Given the geographical proximity between Morocco and Spain and the import trade of tomato fruits between both countries, the introduction of an ancestor of Group 2 from the El Jadida region is a plausible origin for its appearance in Spain.

The TYLCV-Mu15 clone synthesized in this work was infectious. Using this clone we were able to reproduce the symptoms of the disease in susceptible plants, but it was not possible to reproduce the disease in resistant plants. With the exception of a mild mosaic observed at 18 dpi and a slight reduction in size compared to plants inoculated with the empty vector, no other symptoms could be observed in infected *Ty-1* tomato plants. Importantly, similar results were obtained with the TYLCV-IS76 clone in this and other experiments [27]. Thus, under experimental conditions, isolates TYLCV-IS76 and TYLCV-Mu15 appeared not to be able to induce disease in *Ty-1* plants. Trials carried out to evaluate resistance levels for different genotypes concluded that conditions that may occur in summer, such are high inoculum pressure, plant age, high temperatures, and high light intensity may exert very important effects favoring the expression of the disease symptoms [2,46]. Climate also conditions the vector populations, which are favored by low rainfall, high temperatures, and humidity [43,47]. In recent years, in many Mediterranean areas, mild winters and warm springs have resulted in unusually high *Bemisia tabaci* populations early in the year; also, summer temperatures have been unusually high for prolonged periods during recent years. It might be that these conditions, combined with higher TYLCV-Mu15 and TYLCV-IS76 in planta multiplication efficiencies, are responsible for the aggressive symptoms observed in *Ty-1* tomato crops around the Mediterranean. It would be interesting to conduct experiments reproducing extreme summer environmental conditions occurring in the field to test this hypothesis. However, the implication of other unknown factors, including cryptic pathogens such as *Southern tomato virus* (STV) [48], cannot be excluded.

The in planta fitness assays described by Belabess et al. (2016) [27] and in this work, showed that the recombinant isolate TYLCV-IS76 depressed the accumulation of TYLCV-IL or TYLCV-Mu15 in mixed infections. In fact, Belabess et al. (2015; 2016) [27,31] postulated that the greater accumulation of TYLCV-IS76 in mixed infections is consistent with a positive selection of TYLCV-IS76-like isolates that has led to the displacement of their parental viruses in Morocco [27,31]. Also, these authors showed that the ability of TYLCV-IS76 to depress the accumulation of TYLCV-IL in mixed infections depended on the genomic region acquired from TYLCSV through recombination [27]. To date, the presence of isolates similar to the recombinant TYLCV-IS76 or other similar recombinants has not been described in Spain, but if an introduction of a similar isolate occurs, a displacement of the resident TYLCV-IL isolates could be expected. However, due to the presence of Group 2 isolates in Spain, the situation might be more complex, and could be dependent on the frequency of mixed infections (i.e., transmission opportunities), as the accumulation of TYLCV-Mu15 was significantly higher in single infections than that of TYLCV-IS76. This is particularly interesting in light of the data obtained when estimating the differences between synonymous and nonsynonymous substitutions between pairs of isolates, where the calculated value for the C3 gene suggests that positive selection is acting on the Spanish population studied here. This gene encodes a replication enhancer protein, which influences the expression of symptoms by increasing the amount of double and single stranded viral DNA [9]. It is possible that the mutations in C3 are contributing in a decisive way to improve the replication of the virus in tomato cultivars that express the versions of RDRs encoded by *Ty-1*/*Ty-3* [21]. To test this hypothesis, it would be interesting to build a chimeric clone with a Group 1 isolate and compare its accumulation in resistant plants to that of TYLCV-Mu15. In any case, it seems that the ability of TYLCV isolates to win in competition experiments depends on a different genetic determinant (IS76 region) than their ability to accumulate to the highest levels (perhaps C3) in single infections.

We have witnessed the spread and replacement of viruses and strains implicated in the TYLCD throughout the years in Spain. Results shown and discussed here suggest that new changes in the populations of viruses associated with TYLCD in Spain are taking place, with potential complex interactions between viral types, both individually and ecologically.

## 4. Materials and Methods

### 4.1. Samplings

Surveys were conducted during 2015 and 2016 in Águilas and Mazarrón, the most important tomato producing areas of the Region of Murcia (Southeast Spain). Resistant tomato plants showing aggressive TYLCD symptoms were sampled. In 2015, the samplings were carried out in 5 plots from each geographical area, taking 2 samples per plot (Table 1). In 2016, the same areas were sampled again, although in this case the number of plots was 4 per geographical area and 5 samples were taken per plot (Table 1). The samples were taken from the youngest leaves of each plant and the plant material was frozen at −80 °C until used.

### 4.2. DNA Extraction and Geminivirus Detection by PCR

The extraction of DNA from the samples was carried out according to Doyle (1991) [49]. Briefly, 0.06 g of leaf material was placed in a 1.5 mL tube and 600 μL of CTAB extraction buffer (5 M NaCl, 1 M Tris-HCl pH 8, 0.5 M EDTA pH 8, 2% CTAB) and two tungsten spheres were added. The plant material was crushed in a TyssueLyser II (Qiagen, Hilden, Germany) at 30 Hz for 2 min. Next, the samples were incubated at 65 °C for 30 min and then 600 μL of chloroform: isoamyl alcohol (24:1) was added and vortexed until emulsified, followed by centrifugation for 15 min at 2370× *g*. The aqueous phase was taken and mixed with 500 μL of cold isopropanol; the samples were then centrifuged for 10 min at 16,060× *g* and the supernatant was removed. The precipitated DNA was washed with 200 μL of 70% ethanol and finally dissolved in 50 μL of sterile H_2_O and the samples were stored at −20 °C. The geminivirus detection in these extracts was done using degenerate primers that amplify a 580 bp fragment within the CP gene [50]. PCR conditions: Initial denaturation at 95 °C for 2 min, followed by 35 cycles lasting 30 s at 95 °C, 30 s at 53 °C, and 18 s at 72 °C, and a final extension of 5 min at 72 °C. PCR was performed using the GoTaq^®^ DNA Polymerase enzyme (Promega, Madison, WI, USA) and each reaction contained 1xGoTaq^®^ flexi buffer, 1 mM MgCl_2_, 0.2 mM of each dNTP, 200 nM of each primer, 50 ng of template DNA, and 1 U μL^−1^ of the polymerase in a final volume of 25 μL. The PCR products were analyzed by electrophoresis in 1% agarose gels in 1% TAE buffer.

### 4.3. Sequencing

The samples were enriched in circular DNA using the RCA technique [51]. This technique uses phage polymerase Phi29 (TempliPhi. GE Healthcare, Chicago, IL, USA) and random primers to amplify circular DNA molecules, and was carried out following the protocol recommended by the manufacturer. In 2015, the products amplified by RCA were used directly for sequencing by HTS. These were sent to StabVida (Monte da Caparica, Portugal), where after checking the integrity and quality of the DNA, libraries were prepared using the Illumina Nextera XT kit and sequenced with an Illumina-Miseq apparatus generating paired 300-bp readings (Illumina, Inc., San Diego, CA, USA). The bioinformatics analysis of the generated data was carried out using the CLC Genomics 8.5.1 platform (CLCbio, Boston, MA, USA).

For 2016 samples, the RCA products were digested with *Nco*I or *Sac*I (New England Biolabs, Ipswich, MA, USA), digestion products were purified after electrophoresis on 1% agarose gels with the UltraClean^®^ DNA Purification Kit (MO BIO Laboratories, Inc., Carlsband, CA, USA), and cloned into pGEM^®^-T easy vectors (Promega, Madison, WI, USA) digested with the same enzymes and dephosphorylated (Antarctic phosphatase. New England Biolabs, Ipswich, MA, USA) to prevent their re-ligation. *Escherichia coli* Stellar™ Competent Cells (Clontech, Mountain View, CA, USA) were transformed with the ligation products and grown on solid LB medium supplemented with ampicillin (100 mg L^−1^) for selection and Chromomax™ IPTG/X-gal solution (Thermo Fisher Scientific, Waltham, MA, USA) to identify colonies with inserts by white/blue colony discrimination. After culturing the bacteria in liquid medium supplemented with ampicillin, the plasmids were extracted using the Gene JET Plasmid Miniprep Kit (ThermoScientific, Waltham, MA, USA) following the manufacturer’s instructions and sequenced. Sequencing was performed using M13 primers, as well as specific internal primers designed from the isolates sequenced in 2015. The sequences determined in this work have been deposited in the NCBI database with accession numbers MH644778 to MH644788 and MH680947 to MH680958.

### 4.4. Analysis of Sequences

A search for complete sequences in the NCBI database was performed using the BLAST tool [52]. Nucleotide identities between sequence pairs were calculated using the SDT program v1.2 [32]. In this analysis, we included sequences from the seven strains that make up the TYLCV species, and a sequence from a Spanish isolate of the TYLCSV species.

A set of 60 complete sequences was generated including the complete sequences determined in this work and representative sequences of the TYLCV strains, which was edited manually and aligned with the MUSCLE tool included in MEGA7 [53]. In addition, a second set of sequences was generated with those obtained in this work, which was also edited and aligned with MEGA7 [53]. A phylogenetic analysis was carried out with both sets of sequences for which maximum likelihood phylogenetic trees were constructed using the Tamura-Nei TN93 + G model [54], chosen as the best nucleotide substitution model according to MEGA7 [53] and a bootstrap of 1000. Trees were edited using the TreeGraph2 program [55], where the branches with a bootstrap value lower than 50% were collapsed.

An additional alignment was made with Spanish partial sequences available in the databases [16], the sequences obtained in this article, using MEGA7 [53]. With this set of 61 sequences, another phylogenetic analysis was carried out in which a phylogenetic tree of maximum likelihood was constructed with the Tamura-3 parameters T92 + G model [56], chosen in this case as the best model of nucleotide substitution by MEGA7 [53] and a bootstrap of 1000. A tree of partial sequences was constructed with the same model of phylogenetic reconstruction but including only the sequences determined in this work.

A Bayesian analysis was performed using the Beast v.1.8.4 program [57]. A set of 54 sequences was constructed including the 14 sequences of the recombinant TYLCV-IS76 included in Belabess et al. (2015) [31], where all the sequences were trimmed to eliminate the recombined region from TYLCSV. For each sequence, the collection year was annotated for the analysis. The substitution model chosen for the analysis was the same as that used for the maximum likelihood analysis; the Tamura-Nei TN93 + G model [54]. A relaxed uncorrelated lognormal distribution was used as molecular clock and the demographic model constant population size was used as the coalescent prior. The MCMC run was created with 70 million steps in the Markov chain and sampling every 7000 steps. Results were analyzed using Tracer [58]. The resulting tree was constructed using treeAnnotator (available in the BEAST package) and edited using FigTree v1.4.3.

Genetic distances between and within populations were estimated using the Kimura 2-parameter method [59] implemented in MEGA7 [53], and the calculation of the ratio of synonymous and nonsynonymous substitutions between sequence pairs was carried out using the Pamilo–Bianchi–Li method [35,36]. For both calculations, the associated standard error was computed with the bootstrap method with 500 replicates.

An analysis was also carried out to find out possible recombination patterns in the new sequences with the RDP program [60], using the default program parameters.

### 4.5. Infectious Clone

A sequence of 1.1 copies of Mu 5.2bAG: 15 (Table 1) was synthesized by Genscript Inc. (Piscataway, NJ, USA) and introduced into the binary vector pBIN61. This construct was used to transform *Agrobacterium tumefaciens* GV3101. The resulting clone was named pTYLCV-Mu15. To test if this clone could initiate virus infections in plants, two methods were used: direct agroinoculation from the colonies grown on solid media and agroinfiltration from liquid culture. For the first method, transformed bacteria were grown for 48 h at 28 °C in solid LB medium supplemented with kanamycin and rifampicin (both at 50 mg L^−1^). Two to four colonies were then taken with a wooden stick and spread onto the plant surface to be inoculated and, with a hypodermic needle, the area was pricked 3–5 times to introduce the bacteria into the plant. The inoculations were made at the base of the stem (crown), middle zone of the stem, and base of the leaves [7]. For the second method, from the solid culture described above, a preculture (3–5 mL) was started in YEB medium with kanamycin and rifampicin (both at 50 mg L^−1^) which was left stirring at 28 °C overnight. This preculture was transferred to a flask with the same medium and grown under agitation at 28 °C to the desired optical density (0.6–0.8). The culture was then centrifuged at 3894× *g* for 15 min and resuspended at the same optical density (0.6–0.8) in SIM medium supplemented with acetosyringone (200 µM) and incubated for 4 h at 25 °C prior to agroinfiltration. Agroinfiltration with liquid medium was carried out in two ways: following the same scheme as for solid medium but injecting the liquid medium and through agroinfiltration on the underside of the leaves. The infectious clone was evaluated by the agroinoculation of cv. Moneymaker (susceptible) tomato plants using a total of 24 plants, of which 10 were agroinoculated from solid culture, 10 were agroinoculated from liquid culture, and four were left uninfected, as negative controls. After preliminary experiments, the method of pricking colonies and injection was chosen for further tests. The agroinoculation experiments were carried out in plant growth chambers, with conditions of 16 h of light and 8 h of darkness, at a temperature of 25 °C and a relative humidity of 80%.

### 4.6. Virus Accumulation Test

The clones used in this experiment were pTYLCV-Mu15, described above, a clone of TYLCV-IS76 obtained from the reference sequence LN812978 and a clone of TYLCV-IL, prepared from the reference sequence AM40920, both cloned in pCAMBIA0380 and introduced in the *Agrobacterium tumefaciens* C58 MP90 strain, as described by Belabess et al. (2016) [27]. The clones were used both in single and in mixed infections with the preparation of the inocula as described in Belabess et al. (2016) [27]. For the experiment, a total of 92 plants were used, of which 44 belonged to the resistant cultivar Pristyla (*Ty-1*) and 48 to an isogenic susceptible line (Gautier Semeces^®^, Eyragues, France) (Table 3). The agroinoculation was carried out through the agroinfiltration of the cotyledons one week after transplanting. The agroinoculated plants were placed in a growth chamber, with 14 h of light at 26 ± 2 °C and 10 h of darkness at 24 ± 2 °C and irrigated with water fertilized with a solution 15:10:30 N:P:K + trace elements, until the end of the experiment. To minimize the effects of the position in the chamber on the different treatments, the plants were arranged in a random manner. The plants were sampled at 10 and 30 dpi. The youngest adult leaf was identified, and a sample was taken from the central region of each leaflet (five discs per plant). DNA extraction was performed following the protocol of Dellaporta et al. (1983) [61], with the modifications described in Urbino et al. (2013) [62].

Virus accumulation was measured with qPCR. For this, three pairs of primers were used for the three viruses and another pair for the gene coding for the 25S ribosomal RNA, which was used as the endogenous control for normalization (Table 4). Dilutions (1/100) were made from the total DNA extracts and were used for quantification by qPCR. Each reaction contained 2× LightCycler 480 SYBR Green I Master, (Roche, Basilea, Switzerland), 10× of the corresponding primer pair, 2 μL of the sample dilution, and water to a final volume of 10 μL. The qPCRs were perfomed in 384-well plates in a LightCycler 480 thermal cycler (Roche, Basilea, Switzerland) and two technical replicates were done per sample. The concentrations of the primers were 800 nM for pairs that amplified TYLCV-IL and 300 nM for pairs that amplified TYLCV-IS76, as described in Belabess et al. (2016) [27]. The optimal concentration was determined for the pair that amplified the TYLCV-Mu15 DNA, which was also 300 nM. PCR conditions: Initial denaturation of 10 min at 95 °C, followed by 40 cycles of 10 s at 95 °C, 40 s at 63 °C for TYLCV-IL and TYLCV-Mu15, 20 s at 60 °C for TYLCV-IS76 and 25S, and 15 s at 72 °C. The viral genome copy number was calculated from standard curves made with dilutions of the plasmids in base 10 (10^9^–10^2^ copies). The fluorescence data obtained were analyzed with the LinReg program [63], which calculates the initial concentration of the target per sample (*N_o_*) from the fluorescence threshold for each target (*F_t_*), the average of the efficiency of the PCR (*E_mean_*) and the number of cycles necessary to reach that threshold (*C_t_*). The *N_o_* values were transformed from fluorescence values to DNA copy number.

### 4.7. Statistical Analyses

Statistical analyses were performed using the Statgraphics plus 5.1 program (Manugistics, Inc., Rockville, MD, USA). To determine the statistical significance of the virus accumulation data between treatments at 10 and 30 dpi, expressed as the logarithm of no. of copies, and the measurements of the plants made at 30 dpi, the Kluskal–Wallis test was used. To determine the statistical significance of the differences between treatments for the same clone (simple and mixed infections), the Wilcoxon test was used.

## Figures and Tables

**Table 1:** Field sampling.

Tomato Cultivar ^a^	Number of Samples	Sequence Name ^b^	Year	Location	Field
ND	2	Mu 1.1 MZ:15	2015	Mazarrón	1
Boludo	2	Mu 2.1 MZ:15/Mu 2.2 MZ:15	2015	Mazarrón	2
ND	2	Mu 3.1 MZ:15/Mu 3.2 MZ:15	2015	Mazarrón	3
ND	2	Mu 4.2 MZ:15	2015	Mazarrón	4
ND	2	Mu 5.2 MZ:15	2015	Mazarrón	5
Patriarca	2	Mu 1.1 AG:15	2015	Águilas	1
Boludo	2	−	2015	Águilas	2
Cecilio	2	Mu 3.1 AG:15/Mu 3.2 AG:15	2015	Águilas	3
Boludo	2	Mu 4.1 AG:15/Mu 4.2 AG:15	2015	Águilas	4
Patriarca	2	Mu 5.1 AG:15 Mu 5.2a AG:15/Mu 5.2b AG:15	2015	Águilas	5
Jawara	5	Mu 1 MZ:16	2016	Mazarrón	1
Boludo	5	Mu 2 MZ:16	2016	Mazarrón	2
Boludo, Duratom	5	Mu 3 MZ:16	2016	Mazarrón	3
Ramyle, Boludo	5	Mu 4 MZ:16	2016	Mazarrón	4
Jawara, Patriarca	5	Mu 1 AG:16	2016	Águilas	1
Boludo, Jawara	5	Mu 2 AG:16	2016	Águilas	2
Myla	5	Mu 3 AG:16	2016	Águilas	3
Grandoly	5	Mu 4 AG:16	2016	Águilas	4

**^a^** Resistant plants showing TYLCV symptoms; ND: No cultivar information available; ^b^ Sequences are named as Mu field number locality: year. In bold, samples characterized by RCA-HTS.

**Table 2:** Nucleotide diversity values for the different TYLCV genes, calculated using the Pamilo–Bianchi–Li method [35,36]. Results are expressed as mean ± standard error.

Gene	dn	ds	dns − ds
*V1*	0.006 ± 0.002	0.057 ± 0.014	−0.051
*V2*	0012 ± 0.004	0.03 ± 0.013	−0.018
*C1*	0.005 ± 0.001	0.014 ± 0.004	−0.009
*C4*	0.005 ± 0.003	0.009 ± 0.008	−0.004
*C2*	0.006 ± 0.002	0.012 ± 0.006	−0.006
*C3*	0.013 ± 0.004	0.005 ± 0.004	0.008

**Table 3:** Design of the in planta fitness assay.

Cultivar	Treatment ^a^	Num. Infected Plants
Pristyla (*Ty-1*/*ty-1*)	Mu15	11
	IL	6
	IS76	6
	Mu15 + IS76	12
	IL + IS76	6
	Mock ^b^	3
Susceptible (*ty-1*/*ty-1*)	Mu15	11
	IL	7
	IS76	7
	Mu15 + IS76	8
	IL + IS76	12
	Mock ^b^	3

**^a^** Plants agroinoculated with: TYLCV-Mu15 (Mu15), TYLCV-IL (IL), and TYLCV-IS76 (IS76), for single infections; TYLCV-Mu15 + TYLCV-IS76 (Mu15 + IS76) and TYLCV-IL + TYLCV-IS76 (IL + IS76), for mixed infections. ^b^ Plants agroinoculated with an empty vector pCAMBIA 0380.

**Table 4:** Primers and real time PCR conditions used in the fitness assay.

Clone (GenBank acc.)	Primer	Sequence (5′-3′)	Amplicon Size (bp)	Temperature (°C)	Reference
TYLCV-IL (AM409201)	Il-fw	AATGGCTATTTGGTAATTTCG	146	63	[27]
	Il-rev	CGTCTGTGGAACCCTCG			
TYLCV-IS76 (LN812978)	IS76-fw	CCGATAAAGTAGTAGGCCCTACGCA	135	60	[27]
	IS76-rev	AGTGGGTCCCACATATTGCAAGAC			
pTYLCV-Mu15 ^a^	Mu-fw	AATGGCTATTTGGTAATTTCG	146	63	[27]
	Mu-rev	CGTCTGTGGAACCCT**A**G			
Tomato gene 25S RNA ^b^	25S-fw	AGAACTGGCGATGCGGGATG	161	60	[27]
	25S-rev	GTTGATTCGGCAGGTGAGTTGT			

**^a^** Same primers for TYLCV-IL but the reverse was modified (bold letter); ^b^ Endogenous gene used for sample DNA normalization.

## Abbreviations

TYLCD	Tomato yellow leaf curl disease
TYLCV	*Tomato yellow leaf curl virus*
RCA	Rolling Circle Amplification
HTS	High throughput Sequencing
NCBI	The National Center for Biotechnology Information
BLAST	Basic local alignment tool
MUSCLE	Multiple Sequence Comparison by Log-Expectation
MEGA	Molecular Evolutionary Genetic Analysis
SDT	Sequence demarcation tool
RDP	Recombination detection program
MCMC	Markov Chain Monte Carlo
YEB	Yeast Extract Broth
SIM	Simplified Induction Medium
dpi	Days post inoculation

## Appendix A

**Table A1:** HTS results summary for samples collected in 2015 ^a^.

Sample	Num. of Reads ^b^	Mapped Reads	Average Coverage	% of Reference Covered ^c^
Mu 1.1 MZ:15	20,962	63	5.03	97
Mu 2.1 MZ:15	19,394	64	5.5	100
Mu 2.2 MZ:15	19,822	7895	642	100
Mu 3.1 MZ:15	20,322	26	1.93	79
Mu 3.2 MZ:15	18,328	1901	152.44	100
Mu 4.2 MZ:15	43.27	44	3.59	92
Mu 5.2 MZ:15	13,452	358	358	100
Mu 1.1 AG:15	12,424	2202	203.86	100
Mu 3.1 AG:15	31,354	8560	693.27	100
Mu 3.2 AG:15	22,558	133	10.13	95
Mu 4.1 AG:15	253	2218	156.49	100
Mu 4.2 AG:15	20,269	1775	128.37	100
Mu 5.1 AG:15	17,316	1983	163.93	100
Mu 5.2a AG:15	16,302	3848	332.42	100
Mu 5.2b AG:15 ^d^	16,302	2471	199.45	100

^a^ Bioinformatics analysis were performed with *CLC Genomics 8.5.1 workbench*. ^b^ Paired-end reads. ^c^ Percentage of reference sequences covered during de novo assembly. ^d^ Sample used for the construction of the infectious clone TYLCV-Mu15.
